# Spatio-temporal evolution and driving forces of habitat quality in Guizhou Province

**DOI:** 10.1038/s41598-023-33903-8

**Published:** 2023-04-27

**Authors:** Bo Xie, Mingming Zhang

**Affiliations:** 1 College of Forestry, Guizhou University, Guiyang, 550025 Guizhou China; 2 Research Center for Biodiversity and Nature Conservation, Guizhou University, Guiyang, China

**Keywords:** Ecology, Ecology

## Abstract

This study aimed to analyze spatio-temporal changes in habitat quality in Guizhou Province during the 1990–2018 period and identify factors influencing habitat quality. Land-use data for the period were used to evaluate spatio-temporal variations in habitat quality using the InVEST model, and factors influencing habitat quality were analyzed using GeoDetector. According to the results, cultivated land and forestland decreased by 0.48% and 0.88%, respectively, during the study period. Grassland, water, and construction land areas increased, with construction land increasing the most (0.92%) followed by water area (0.37%). The main land-use changes included conversion of cultivated land to forestland, grassland, and construction land. The average habitat quality index for Guizhou Province changed from 0.633 to 0.627 over the 1990–2018 period, showing an overall downward trend. The distribution pattern of habitat quality was spatially "high in the north, south, and, east, and low in the west". The most significant improvement of habitat quality was in the western region, whereas the most significant decline of habitat quality was in the central region. Land-use was the major factor influencing the spatio-temporal variations in habitat quality, and the interactive effect between any two factors was stronger than that of a single factor. Natural factors and human factors co-dominated the temporal-spatial changes in habitat quality.

## Introduction

Habitat quality (HQ) refers to the ability of an ecosystem to provide suitable conditions for the survival of organisms, and is a prerequisite and basis for ecosystem functions and services^1^. HQ reflects the suitability of an environment for human survival, reproduction, and productivity^2^. In addition , it is a key indicator of ecological health, which is essential for human well-being^3,4^. Land is the carrier of habitat and land-use change is a primary manifestation of the impact of human activities on the land surface, including changes in proportions, structure, and intensity of land-use. Land-use changes fundamentally alter HQ and the composition and structure of ecosystems, which affects energy flow and nutrient cycling between habitat patches^5,6^. The increase in land-use changes and structure has led to habitat fragmentation, degradation or even habitat loss, in turn, resulting in a continuous decline in HQ^7^.

HQ is a significant foundation for ecosystem service capacity and biodiversity maintenance^8^. Generally, methods for assessing HQ at the landscape scale fall into two categories^9^. The first assessment method is based on field surveys and is mostly applicable at small scales. The survey-based assessment method is time-consuming, laborious, and obtaining long-term species data is a challenge, often unable to assess the spatio-temporal dynamics of biodiversity^10^. The second assessment method is based on ecological assessment models, such as the habitat suitability index (HIS) model^11^, integrated valuation of ecosystem services and trade-offs (InVEST) model^6^, and social values for ecosystem services (SolVES) model^12^ to assess regional HQ. Among these models, the InVEST model is currently the most developed and extensively applied ecological function assessment model^13^. InVEST model is a geographic information system-based model that can rapidly evaluate the impact of various land-use changes and threats to biodiversity^3^. The results obtained by the InVEST model are more spatially visualized^14^ and can reflect the distribution of habitats and their degradation in different landscape types. The InVEST model has recently been used to evaluate the impact of land use and land cover (LULC) changes and protection tactics on habitats to sustain biodiversity^15^. Existing studies have mainly assessed HQ in mountainous areas^16^, watersheds^17^, nature reserves^18^, counties^4^, urban agglomerations^8^, and provinces^19^. However, most studies at the provincial scale have focused on the spatio-temporal evolutionary characteristics of HQ^20,21^, with few studies investigating the factors influencing HQ.

HQ is the ability of an ecosystem to support species survival^22^. High quality habitats support a rich biodiversity, whereas low quality habitats denote poor biodiversity^23^. In recent years, HQ changes based on LULC changes have been extensively studied^24,25^. For example, the expansion of urban areas has severely damaged natural habitats, leading to biodiversity loss^26,27^. In addition, natural factors (altitude, slope, and climate) and land-use types substantially influence HQ changes^28,29^. Therefore, determining factors that influence HQ could provide basic information for biodiversity maintenance, improvement of the ecological environment, and enhancement of ecosystem services and functions^30^. Although previous research has focused on the spatiotemporal evolution characteristics and influencing influencing HQ, further studies regarding the subject are required. The main methods used to explore the factors influencing HQ include correlation analysis^31^, ordinary least squares model (OLS) and geographically weighted regression model (GWR)^32^ model, GeoDetector^33^, and other statistical models. However, correlation analysis, OLS and GWR cannot explore the effects mechanism of multiple factors on the spatial pattern of HQ. GeoDetector is a widely used spatial analysis tool that can explore the heterogeneity and explanatory power of various factors in space^34^, and is widely used in geography and environmental science. Guizhou Province is an important ecological barrier in the upper reaches of the Yangtze and Pearl Rivers and is crucial to the sustainable development of the Yangtze River Basin. However, few studies have investigated HQ and factors influencing HQ in Guizhou Province.

Guizhou Province is the transportation hub of southwest China and an important part of the Yangtze River Economic Belt. With the rapid development of the economy, various land types have undergone drastic changes, and environmental problems have become increasingly prominent. Moreover, Guizhou Province is one of the first national ecological civilization pilot areas. To effectively implement ecological strategies and accelerate the development of early demonstration zones of ecological civilization, it is crucial to explore the variations in spatio-temporal patterns of HQ. Therefore, this study used the InVEST-HQ model to (1) analyze the spatio-temporal changes in HQ in Guizhou Province during the 1990–2018 period, (2) assess the evolutionary patterns of regional HQ, and (3) identify the factors influencing spatial changes in HQ using the GeoDetector. The results could guide future planning and development of ecological environments, in addition to promoting the development of ecological civilization.

## Materials and methods

### Study area

**Figure 1:**
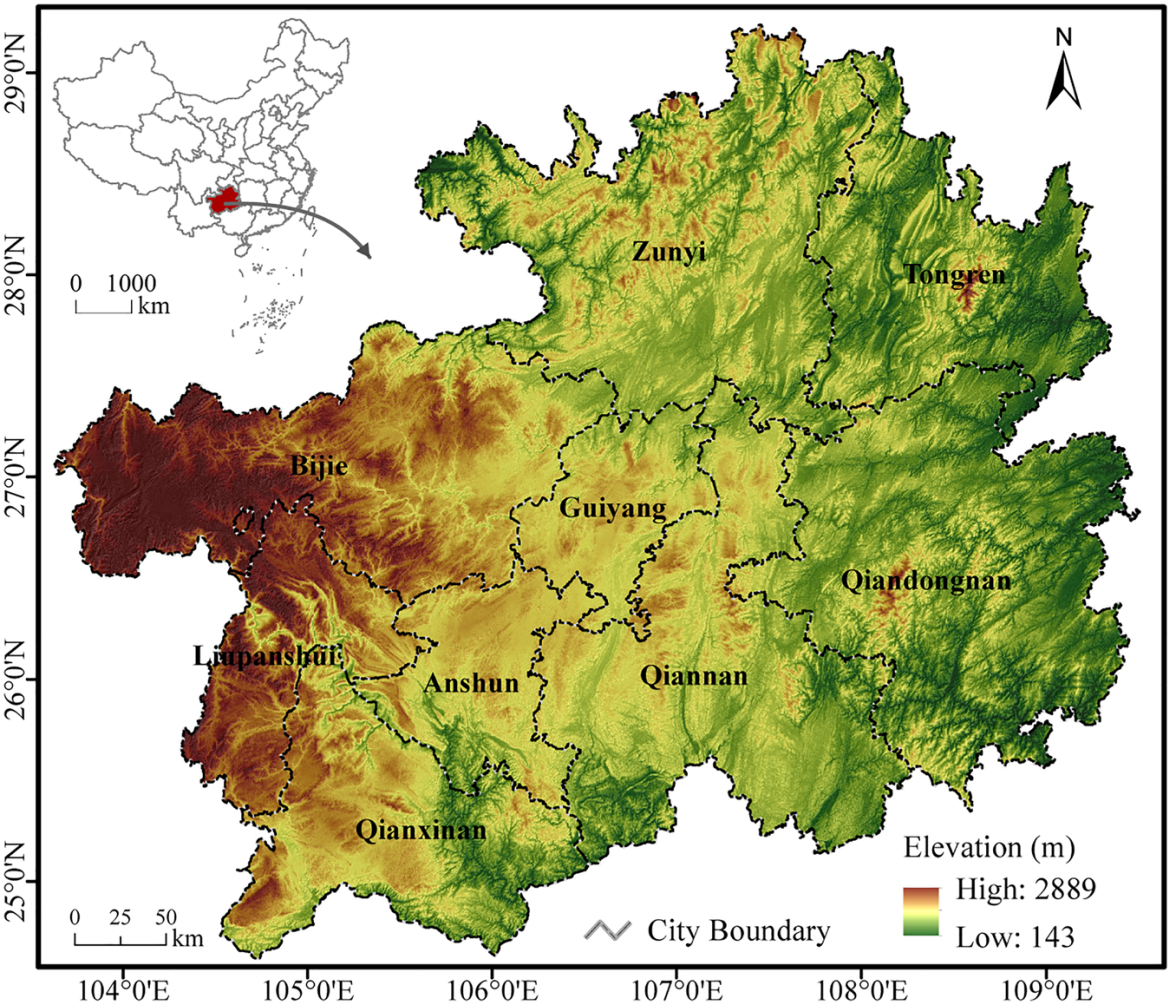
Location of Guizhou Province, China (created by ArcMap, version 10.7, http://www.esri.com/).

Guizhou Province, which is located in the hinterland of southwest China (103°360–109°350 E; 24°37–29°130 N) (Fig. 1), and covers a total area of 1.76 × 10^5^ km^2^. Guizhou Province belongs to the low-latitude zone and experiences a subtropical humid monsoon climate with abundant annual precipitation of 1000–1400 mm and relative humidity is above 70%. The annual average temperature is 15 °C. Guizhou is a unique mountainous province in China without plains, more than 90% of the total area is occupied by mountains or hills with the altitudes ranging from 143 to 2889 m. The complex topography and climate contribute to the diversity of land-use types. The study area has nine municipalities and is quite underdeveloped. The population pressure is generally high, with the province having a population of more than 36 million population in 2019. Majority of the residents are poor and live in poverty in remote mountain villages (Data obtained from *Guizhou Statistical Yearbook*, http://stjj.Guizhou.gov.cn). Guizhou Province is a relatively unique regional environment unit, where over 60% of the total area is covered by an outcrop carbonate karst. The unique geological structure makes the area a vulnerable ecosystem.

### Data sources

Land-use data for 1990, 2000, 2010, and 2018 were obtained from the Data Center for Resources and Environmental Sciences of the Chinese Academy of Sciences (http://www.resdc.cn) and the spatial resolution was 30 m × 30 m^35^. The land-use data included six primary land-use types and 20 secondary land-use types (Table 1). The accuracy of interpretation of the data was more than 90%, which satisfied the requirements of the study^36^. Digital elevation model (DEM) data was acquired from the Geospatial Data Cloud with a resolution of 30 m (http://www.gscloud.cn/) using ASTER global DEM (v2). Slope and elevation were acquired from DEM data. Temperature, precipitation, net primary productivity (NPP), normalized difference vegetation index (NDVI), population density, and gross domestic product (GDP) data were obtained from the Data Center for Resources and Environmental Sciences of the Chinese Academy of Sciences at a resolution of 1 km × 1 km.

**Table 1:** Land use classification system.

Primary land-use type	Secondary land-use type
Cultivated land	Paddy land (11), dry land (12)
Forest land	Forest (21), shrub (22), open forest (23), other forests (24)
Grassland	High-coverage grassland (31), moderate-coverage grassland (32), low-coverage grassland (33)
Water	River canalization (41), lake (42), reservoir and pond (43), beach land (46)
Construction land	Urban land (51), rural settlement (52), other construction land (53)
Unused land	Bare land (63), saline land (64), marshland (65), bare rock (66)

Specifically, temperature and precipitation data were generated using the ANUSPLIN interpolation software. The spatial distribution dataset (NDVI) comprises a monthly vegetation index dataset generated since 1998 based on the SPOT-VEGETATION NDVI satellite remote sensing data of continuous time series and the maximum synthesis method. GDP dataset, which is based on the national GDP data of a county, describes the spatial relationships among land-use type, night lighting brightness, settlement density data closely associated with human activities and GDP, and generates spatial grid data through spatial interpolation. The spatial distribution data for population density is basically consistent with GDP. NPP is the fixed energy or organic matter produced per unit area and time left after a green plant respires, and is based on simulations of the light energy utilization model, GLO-PEM. In addition, due to the availability of data, NPP, temperature, precipitation, and population density data were from 2015; while GDP and NDVI data were from 2018.

## Methods

### Geological atlas analysis

Geographical Atlas analysis can simultaneously express the spatial dynamic changes in landscape structure over time, as well as synthesizing land-use changes and HQ patterns by mapping land-use and HQ^37,38^. To determine land-use and HQ changes in Guizhou Province over the 1980–2018 period, growth map, decrease map, and HQ grade transfer map of land-use changes for the period were obtained using the following formula^39,40^:

1 $$\mathrm{N}=10\mathrm{F}+\mathrm{L}$$

where N is the code for land-use change type; F is the code for land-use type before conversion; L is the code for land-use type after conversion.

### InVEST-HQ assessment

The InVEST-HQ model was used to assess HQ of Guizhou Province, which was estimated based on information regarding LULC changes and biodiversity threats^25^. The assessment depended on the distance and intensity of habitat and non-habitat land-use. HQ was reflected by the HQ index that was calculated as follows^22^:

2 $${\mathrm{Q}}_{x\mathrm{j}}={\mathrm{H}}_{\mathrm{j}}\left[1-\left(\frac{{\mathrm{D}}_{x\mathrm{j}}^{\mathrm{z}}}{{\mathrm{D}}_{x\mathrm{j}}^{\mathrm{z}}+{\mathrm{K}}^{\mathrm{z}}}\right)\right]$$

where Q_xj_ denotes the HQ of pixel x in land-use type j, D_xj_ is the threat level of pixel x in land-use type j, H_j_ is the habitat suitability of land-use type j, and k is half the saturation constant (half of the maximum value of D_xj_).

3 $${D}_{xj}=\sum_{r=1}^{R}\sum_{y=1}^{{Y}_{r}}\left({\omega }_{r}/\sum_{r=1}^{R}{\omega }_{r}\right){r}_{y}{i}_{rxxy}{\beta }_{x}{S}_{jr}$$

where *R* denotes the number of stress factors, *Y*_*r*_ is the total number of grid cells of stress factors, *ω*_*r*_ is weight of the stress factor, *r*_*y*_ is the number of stress factors in a grid cell, *β*_*x*_ is the accessibility level of grid *x*, *S*_*jr*_ is the sensitivity of land-use type *j* to stress factors, with a value range of 0–1, and *i*_*rxy*_ is the maximum distance of influence of the stress factors.

4 $${i}_{rxy}=1-\left({d}_{xy}/{d}_{rmax}\right)$$

5 $${i}_{rxy}=1-\left({d}_{xy}/{d}_{rmax}\right)$$

where *d*_*xy*_ denotes the distance between grid *x* and *y*, and *d*_*r*max_ is the maximum impact scope of the threat factor *r*.

The data inputs (spatial and nonspatial), required to run the InVEST-HQ model include multiple-date LULC maps, threat sources and impacts, habitat types, habitat sensitivity to the threats, and the half-saturation constant. InVEST-HQ model finally generates an HQ map with values ranging from 0 to 1^41^, where 1 represents the highest suitability. Construction land reflects the threat to biodiversity threat posed by human activities. As a semi-natural and semi-artificial ecosystem, cultivated land has a negative impact on the natural environment^42^. Therefore, construction and cultivated lands were used as threat factors. Considering that the study area is a typical karst landform, predominantly a mountainous and hilly plateau with scattered rural settlements, construction land was converted to urban construction land, rural settlements, and other types of land-use. Based on the model manual^22^ and literature review^43,44^, the relative weight of threat factors, the maximum distance between habitats and each threat source, and the habitat sensitivity of each threat were determined (Tables 2 and 3).

**Table 2:** Threat factors and maximum effect distances, weights of threat factors, and decay types identified in the study area.

Threats factor	Max distance of influence (km)	Weights	Decay type
Paddy field	1	0.7	Linear
Non-irrigated arable land	1	0.7	Linear
Urban land-use	8	1.0	Exponential
Rural settlements	5	0.6	Exponential
Other construction land	3	0.5	Exponential

**Table 3:** Habitat suitability and sensitivity of land-use types to each threat factor.

Land-use type	Habitat suitability	Paddy field	Dry land	Urban land-use	Rural settlements	Other construction land
Paddy field	0.4	0.0	0.5	0.7	0.6	0.5
Dry land	0.2	0.5	0.0	0.7	0.6	0.5
Forest land	1.0	0.8	0.6	0.6	0.3	0.7
Shrubbery	0.8	0.7	0.7	0.8	0.3	0.7
Open forest land	0.7	0.7	0.8	0.7	0.5	0.6
Other forest land	0.6	0.6	0.7	0.7	0.5	0.6
High coverage grassland	0.8	0.8	0.8	0.5	0.7	0.5
Medium coverage grassland	0.7	0.6	0.6	0.7	0.5	0.3
Low coverage grassland	0.5	0.5	0.5	0.6	0.5	0.4
River canalization	0.8	0.8	0.6	0.5	0.4	0.3
Lake	0.9	0.7	0.7	0.7	0.3	0.5
Reservoir and pond	0.6	0.7	0.6	0.7	0.5	0.5
Beach land	0.5	0.6	0.8	0.7	0.5	0.5
Urban land	0.0	0.0	0.0	0.0	0.0	0.0
Rural settlements	0.0	0.0	0.0	0.0	0.0	0.0
Other construction land	0.0	0.0	0.0	0.0	0.0	0.0
Bare land	0.1	0.1	0.1	0.1	0.1	0.1

### Global Moran’s I

Global Moran’s I is a measure of spatial autocorrelation proposed by Patrick Moran^45^. Spatial autocorrelation refers to the degree to which an attribute of a geographical location is related to different spatial locations, and can be used to describe whether HQ has a clustering effect over the entire area, with a value range of [−1, 1]^46^. Global Moran’s I was calculated using the following formula:

6 $$\mathrm{I}=\frac{\mathrm{n}\sum_{\mathrm{i}=1}^{\mathrm{n}}\sum_{\mathrm{j}=1}^{\mathrm{n}}{\upomega }_{\mathrm{ij}}\left({\mathrm{y}}_{\mathrm{i}}-\overline{\mathrm{y} }\right)\left({\mathrm{y}}_{\mathrm{j}}-\overline{\mathrm{y} }\right)}{\sum_{\mathrm{i}-\mathrm{i}}^{\mathrm{n}}{\left({\mathrm{y}}_{\mathrm{i}}-\overline{\mathrm{y} }\right)}^{2}}$$

7 $${Z}_{score}=\frac{I-E\left(I\right)}{\sqrt{VAR\left(I\right)}}$$

where I is the global Moran’s index; n is the total number of study units; y is the average habitat quality; y_i_ and y_j_ are the habitat quality indices for i and j study areas; w_ij_ is the spatial weight coefficient matrix of regions i and j, which reflects the spatial relationship between regions i and j, and is defined as w_ij_ = 1 or otherwise w_ij_ = 0; and E(I) and VAR(I) represent the expected value and variance of the global Moran’s index y, respectively.

### Hot-spot analysis (Getis-Ord Gi*)

Hot spot analysis is a method of evaluating the distribution of clusters in local areas and can be used to indicate whether there are statistically significant high and low values in the spatial distribution pattern of HQ^47^. A significantly positive G* value shows HQ is a clustering of high values, which is a hot spot, whereas a significantly negative G* value shows HQ is a clustering of low values, which is a cold spot^48^. The G*value was calculated using the following formula:

8 $${G}_{i}^{*}=\frac{\sum_{j=1}^{n}{w}_{ij}{x}_{j}-\overline{x }\sum_{j=1}^{n}{w}_{ij}}{S\sqrt{\frac{{\left[n\sum_{j=1}^{n}{w}_{ij}^{2}-\left(\sum_{j=1}^{n}{w}_{ij}\right)\right]}^{2}}{n=1}}}$$

where xj is the habitat quality of grid j; Wij is the spatial weight matrix of grid i and grid j, if i is adjacent to j, its spatial weight is 1, otherwise it is 0; is the average habitat quality; S is the standard deviation of habitat quality; n is the total number of rasters.

### GeoDetector analysis

GeoDetector is a statistical tool used for detecting spatially stratified heterogeneity and revealing the factors associated with it. The GeoDetector has four main components: divergence and factor detection, interaction detection, risk zone detection, and ecological detection, which are mainly used to analyze the factors influencing various phenomena and the interactions among multiple factors^36,49^. We analyzed the extent to which factors influence spatial variations in HQ using factor and interaction detectors. The factor detector is typically used to measure the extent to which each factor is associated with spatial variation in HQ while the interaction detector is used to measure the interaction between two factors. The magnitude of the divergence is determined by the q-value of the GeoDetector, which is calculated using the following formula^50^:

9 $$\mathrm{q}=1-\frac{\sum_{\mathrm{h}=1}^{\mathrm{L}}{\mathrm{N}}_{\mathrm{h}}{\upsigma }_{\mathrm{h}}^{2}}{{\mathrm{N\sigma }}^{2}}$$

where q is the degree of explanation of the spatial distribution of HQ by an influencing factor with a value ranging from 0 to 1, where larger values indicate a greater effect of the factor on the spatial variation of HQ and smaller values indicate a small effect of the factor; L is the sample size of the influencing factor; N_h_ and N are the numbers of cells in stratum h and the entire area, respectively; σn^2^ and σ^2^ are the variances of HQ in stratum h and the entire area, respectively.

Interaction detection focuses on determining the interaction between various factors and assessing whether multiple factors acting together increase or decrease the driving power of HQ or whether there is no interaction between the factors (Table 4)^50^.

**Table 4:** Types of interactions between two Covariates to HQ.

Judgments based	Interaction
q($x$ 1∩$x$ 2) < min(q($x$ 1),q($x$ 2))	Weaken, nonlinear
Min(q($x$ 1),q($x$ 2)) < q($x$ 1∩$x$ 2) < max(q($x$ 1),q($x$ 2))	Weaken, uni
q($x$ 1∩$x$ 2) > max(q($x$ 1),q($x$ 2))	Enhance, bi
q($x$ 1∩$x$ 2) = q($x$ 1) + q($x$ 2)	Independent
q($x$ 1∩$x$ 2) > q($x$ 1) + q($x$ 2)	Enhance, nonlinear

The study area was sampled based on a 5 km × 5 km grid using ArcGIS 10.7 and a total of 7036 evaluation units were obtained. The factors influencing HQ were divided into natural and human factors, where natural factors included elevation (X1), slope (X2), temperature (X3), rainfall (X4), NPP (X5), NDVI (X6), whereas human factors included population density (X7), GDP (X8), and land-use type (X9). The HQ index was the dependent variable and the factors influencing HQ were the independent variables. In addition, the natural breakpoint method of ArcGIS 10.7 was used to discretize and classify each factor, and correlation results were calculated using the GeoDetector.

## Results

### InVEST-HQ assessment

#### Land-use change analysis

From 1990 to 2018, substantial changes in land-use patterns occurred in Guizhou Province and forestland, cultivated land, and grassland were the main land-use types (Table 5). Grassland, water, and construction land areas increased, whereas cultivated land, forestland, and unused land areas decreased during the study period. Specifically, cultivated and construction land areas increased significantly from 1990 to 2018 by − 98.37% and 398.71%, respectively, followed by grassland and water areas; unused land area was relatively stable. Land-use changes in Guizhou Province were mainly characterized by a significant decrease in forestland and increases in construction land, water, and grassland areas.

**Table 5:** Variations in the areas of different land types in Guizhou Province over the 1980–2018 period.

LULC	1990	2000	2010	2018	Change (km^2^)	Change (%)
Cultivated land	49,285.3	49,653.3	49,413.5	48,434.4	−850.9	−98.27
Forest land	94,641.9	93,526.7	95,515.1	93,102.3	−1539.6	−98.37
Grassland	31,188.8	31,864.5	29,572.8	31,317.1	128.3	100.41
Water	395.1	407.7	689.3	1043.9	648.8	264.21
Construction land	541.7	600.8	874.5	2159.8	1618.1	398.71
Unused land	40.3	40.3	29.8	30.5	−9.8	−75.68

**Figure 2:**
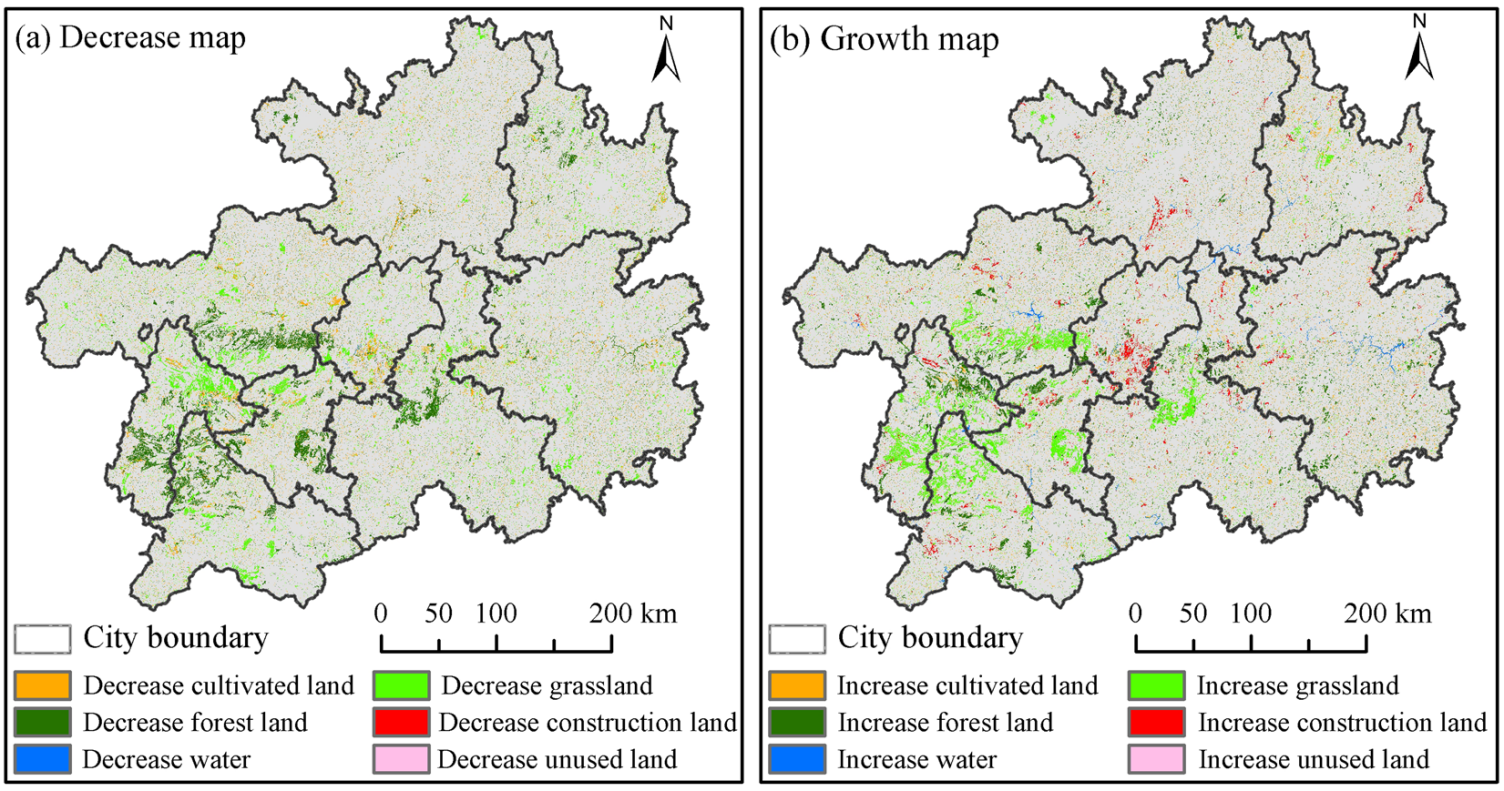
Maps showing the variations in land-use changes from 1980 to 2018 in Guizhou Province.

Maps showing increases and decreases in land-use changes in Guizhou Province from 1980 to 2018 were obtained from a geographic atlas (Fig. 2). Land-use transformations in Guizhou Province during the 1990–2018 period mainly involved cultivated land, forestland, and grassland, as well as the conversion of forestland, cultivated land, and grassland to construction land. Cultivated land was mainly transformed into forestland 3557.52 km^2^, which was sporadically distributed across the province; forestland was transformed into grassland 4707.10 km^2^, which was largely distributed in the central and western regions; grassland was transformed into forestland 3810.20 km^2^, which was distributed in the western region; other land-use types were converted to construction land 1664.16 km^2^, which was distributed in the central region of the study area. Specifically, the conversion of cultivated land during the 1990–2018 period was the most notable land-use change, with 1357.50, 218.67, and 968.39 km^2^ of cultivated land area being converted to grassland, water, and construction land, respectively. A total of 3526.27km^2^, 337.85km^2^, and 371.26 km^2^ of forestland were transformed into cultivated land, water and construction land, respectively. A total of 1689.22, 130.14, and 321.57 km^2^ were transformed into cultivated land, water, and construction land, respectively (Table 6). Therefore, the increase in construction land in Guizhou Province is primarily attributed to the loss of cultivated land, forestland, and grassland, while the decrease in forestland is attributed to the increase in grassland.

**Table 6:** Land-use change transfer matrix of Guizhou Province (km^2^) over the 1980–2018 period.

Land-use type	Cultivated land	Forest land	Grassland	Water	Construction land	Unused land
Cultivated land	43,178.37	3557.52	1357.50	218.67	968.39	0.83
Forest land	3526.27	85,682.44	4707.10	337.85	371.26	2.666
Grassland	1689.22	3810.20	25,230.44	130.14	321.57	1.41
Water	15.52	19.49	7.84	349.88	1.71	0.03
Construction land	21.15	12.86	8.32	3.73	495.59	0.00
Unused land	1.73	8.966	2.76	0.11	1.23	25.53

#### Temporal and spatial variations in HQ

The InVEST-HQ model was used to obtain a HQ map of Guizhou Province and HQ was graded into five levels using the natural breakpoint method as follows: low (0–0.2), moderately low (0.2–0.4), medium (0.4–0.6), moderately high (0.6–0.8), and high (0.8–1.0). The habitat areas and percentages of each grade over the four periods are summarized in Table 7.

**Table 7:** Areas and proportions of HQ grades in Guizhou Province over the 1990–2018 period.

HQ grade	1990		2000		2010		2018	
	km^2^	%	km^2^	%	km^2^	%	km^2^	%
Low	12,117.80	6.88	11,953.34	6.79	12,262.18	6.96	10,960.50	6.22
Medium low	18,900.55	10.73	19,515.50	11.08	19,795.50	11.24	19,935.41	11.32
Medium	61,417.88	34.88	61,902.69	35.15	60,879.72	34.57	61,914.68	35.16
Medium high	38,305.66	21.75	38,436.28	21.83	36,867.16	20.94	37,984.99	21.57
High	45,347.73	25.75	44,281.80	25.15	46,285.02	26.28	45,293.92	25.72
Mean HQ	0.633		0.629		0.630		0.627	

**Figure 3:**
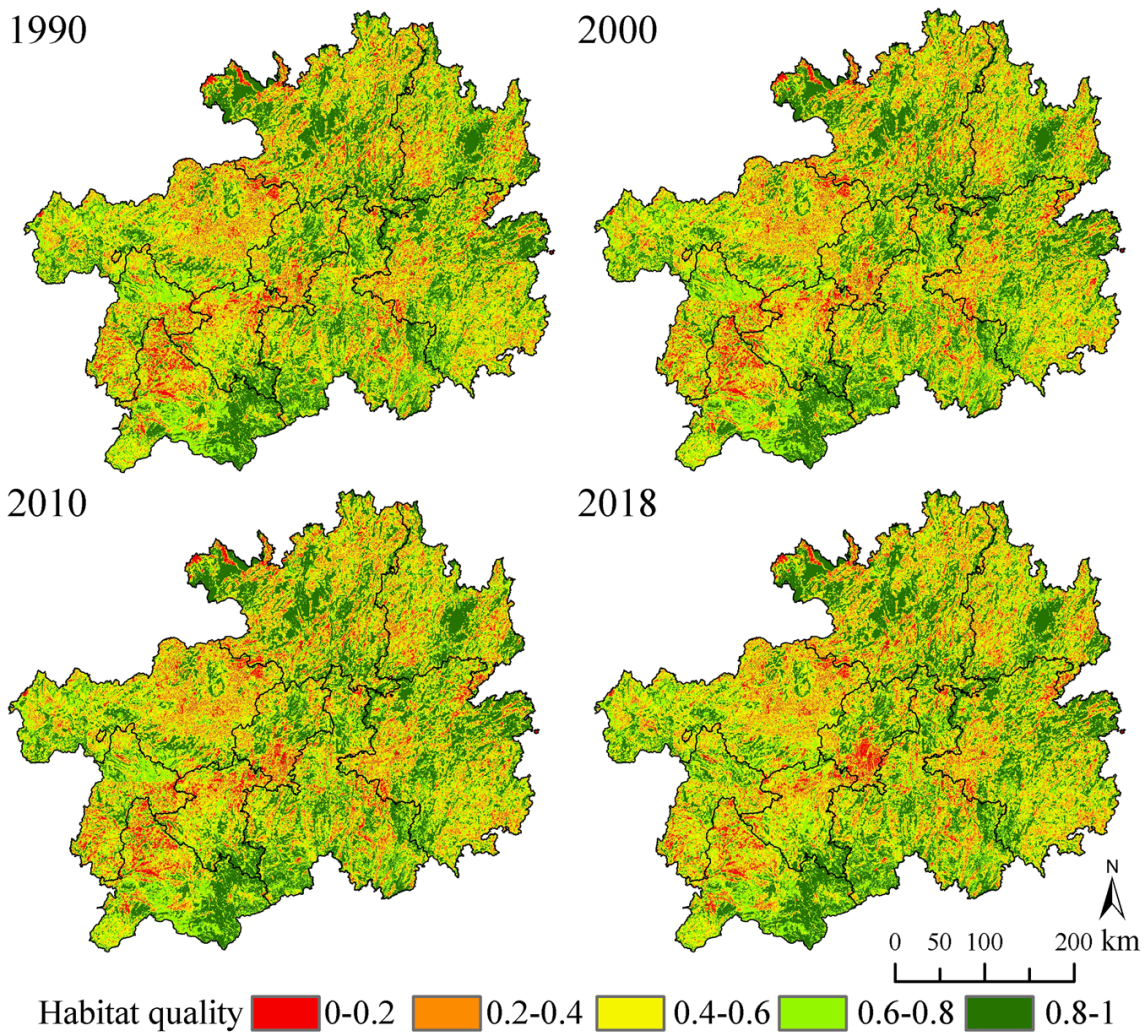
Spatio-temporal patterns of HQ over the 1990–2018 period.

From a spatial scale (Fig. 3). HQ for construction and rural residential land was low, while that for natural forest landscape and grassland was high. The main habitat types in Guizhou Province had medium and high HQ (Table 7). High HQ areas were mainly distributed in the central and western parts of Zunyi, central part of Tongren, and southwestern and eastern parts of Qianxinan. However, low HQ areas were predominantly distributed in the southern part of Guiyang, eastern part of Bijie, and northern parts of Anshun and Qianxinan.

Based on temporal changes, the proportion of low HQ areas decreased by 0.66% (from 6.88 to 6.22%) over the 1990–2018 period; the proportions of both moderately low and medium HQ areas increased by 0.95% (from 10.73% to 11.32%) and 0.28% (34.88% to 35.16%), respectively. The proportions of moderately high and high quality habitat areas decreased by 0.18% (from 21.75 to 21.57%) and 0.03% (from 25.75 to 25.72%), respectively, during the study period (Table 6).

Overall, the average HQ indices for Guizhou Province in 1990, 2000, 2010, and 2018 were 0.633, 0.629, 0.630, and 0.627, respectively, indicating that the HQ declined slightly over the period. HQ initially decreased, then increased, and eventually decreased. The overall habitat change in Guizhou Province was negligible due to the relatively stable proportions of forestland, grassland, and cultivated land that make up the landscape patch of the province. The percentage decreases in HQ over the 1990–2000, 2000–2010, and 2010–2018 periods were 0.63%, 0.15%, and 0.47%, respectively.

**Figure 4:**
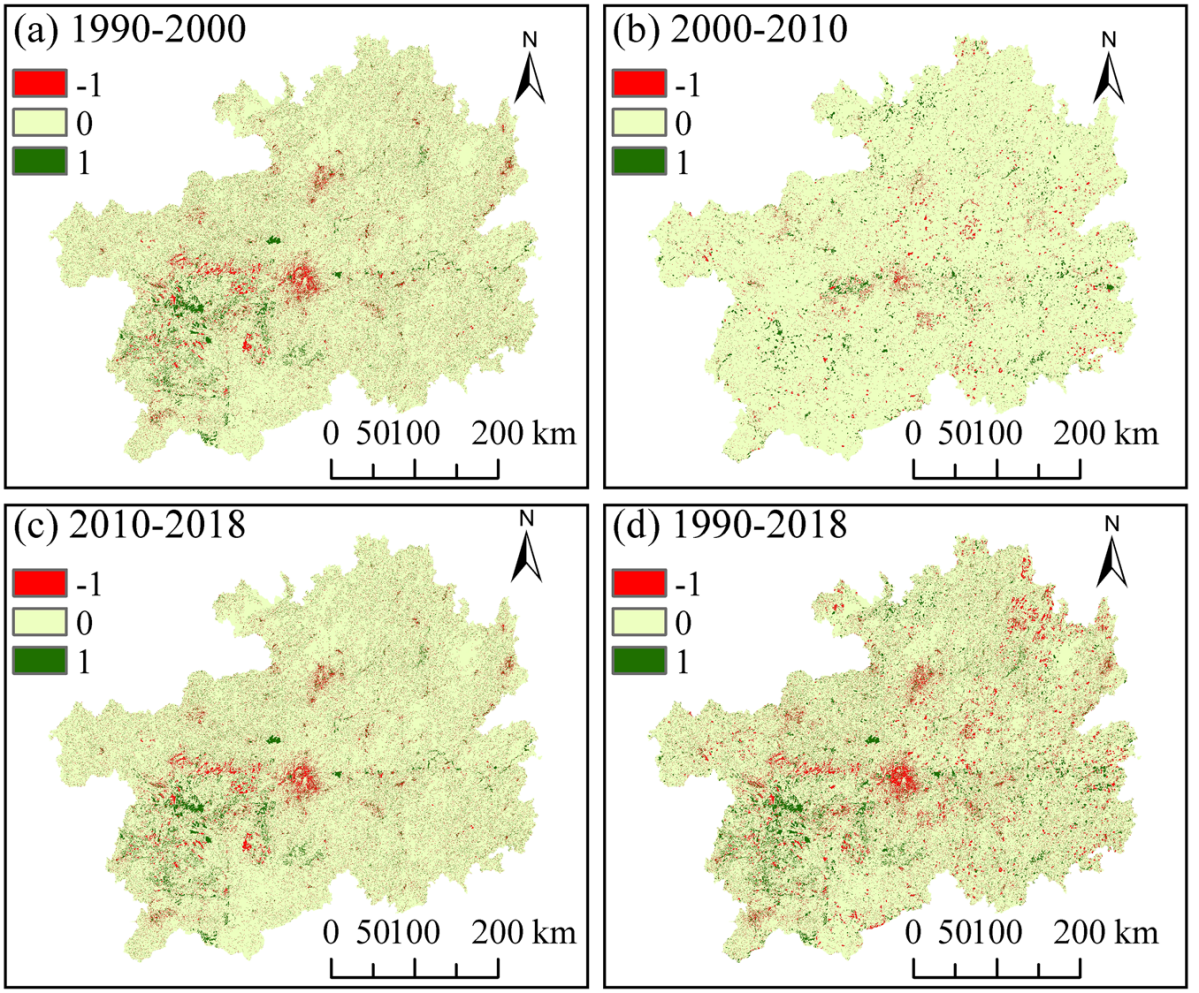
Variations in the spatial patterns of HQ in Guizhou Province over the 1990–2018 period.

To gain insight into the spatial and temporal characteristics of HQ, ArcGIS was used to generate a map showing the spatial variations in HQ in Guizhou Province from 1900 to 2018 (Fig. 4). HQ remained stable in most parts of Guizhou Province from 1990 to 2000, with significant decline in HQ being observed in the central and western regions and increased HQ being observed in the southwestern region. HQ of Guizhou Province improved from 2000 to 2010, although a decrease in HQ was observed in the central region. HQ changed significantly from 2000 to 2010, with a decrease in HQ being observed in areas located in the central-western and northern regions of Guizhou Province, and improved HQ being observed in the southwestern region. Overall, no significant differences were observed in HQ of 84% of the total land area from 1990 to 2018. HQ has decreased in Guizhou Province, especially in the central, western, and northern regions due to the rapid increase in construction land caused by accelerated urbanization, which has in turn, stressed the surrounding habitats and increased habitat fragmentation and loss of connectivity, in addition to decreasing HQ in the areas. The habitats with improved quality were mainly located in the southwestern region, which is attributed to the implementation of projects, such as “returning cultivated land to forestland” and “closing mountains for afforestation and grass cultivation”.

**Figure 5:**
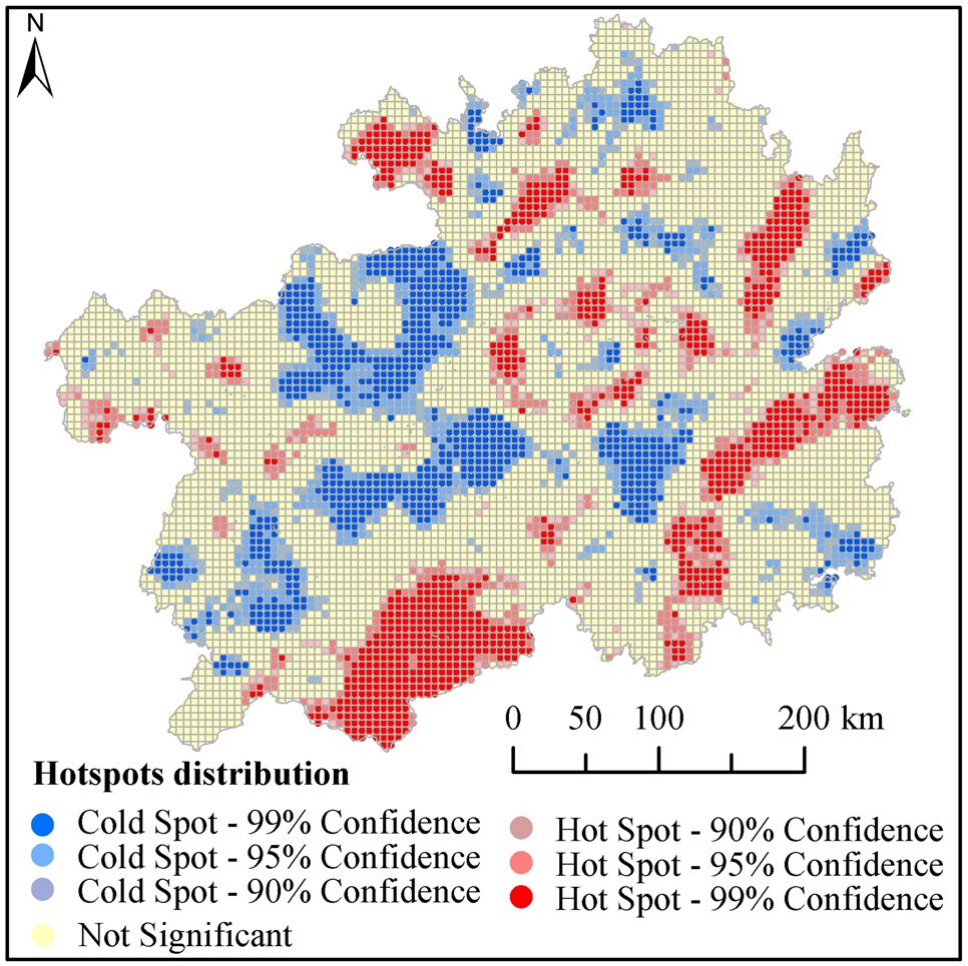
Distribution of hot and cold spots associated with HQ in 2018.

Spatial variations in HQ of Guizhou Province were assessed by performing global Moran’s I statistical test and hot spot analysis. To begin with, global Moran’s I statistic was used to examined the distribution of HQ in Guizhou Province. Global Moran’s I values of HQ in 1990, 2000, 2010, and 2018 were 0.210, 0.206, 0.203, and 0.213, respectively, and P values were 0. The results indicate that HQ had significant spatial agglomeration. An increase in the global Moran's I value in 2018 indicated an increase in the spatial clustering of HQ. Subsequently, hot spot analysis was performed to detect local agglomeration and distribution characteristics of HQ. We analyzed data for 2018 owing to the slight variations in the spatial distribution patterns of HQ during the 1990–2018 period (Fig. 5). The overall geographical distribution of HQ in Guizhou Province was "high in the south and east, low in the central and west, and high and low in the north". The hot spots were mainly concentrated in the southern and eastern regions, where grasses are interspersed in forests, and a few hot spots were distributed in the northern and central regions of the woodland and water areas during the study period. Cold spots were mainly distributed in construction land and some portions of cultivated land.

#### Variations in HQ characteristics of different LULC

**Figure 6:**
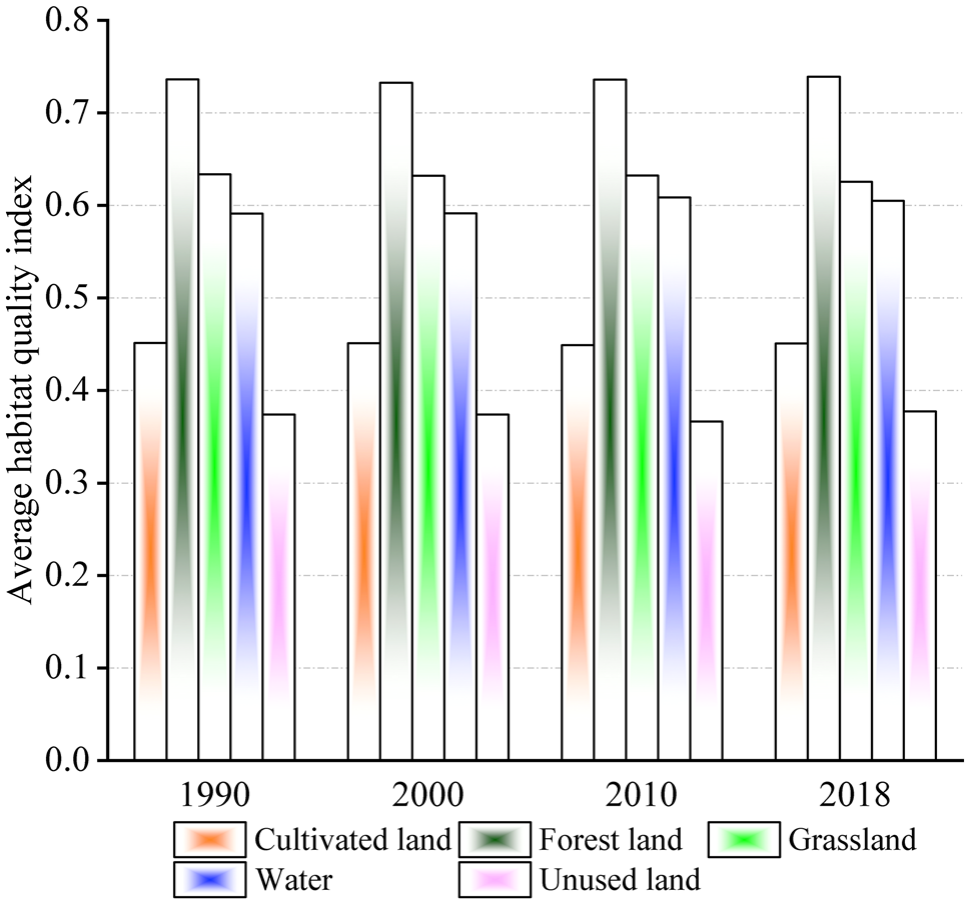
Average HQ indices of different land-use types.

Land-use has a significant impact on regional HQ and changes in land-use lead to changes in HQ^51^. Human activities have altered the spatial patterns of land-use, thereby resulting in habitat change and biodiversity loss. To better understand the impact of land-use change on HQ, the mean values of HQ indices for different land-use types in Guizhou Province in 1990, 2000, 2010, and 2018 were analyzed (Fig. 6).

HQ indices for the different land-use types varied over the four study periods. During the 1990–2018 period, forestland had the highest average HQ index, followed by grassland and water, and the lowest average HQ value was observed in construction land. HQ indices of forestland, cultivated land, and unused land did not exhibit significant variations; however, the HQ index of water initially increased and then decreased. The HQ indices of grassland, cultivated land, and water remained unchanged from 1990 to 2000, while those of forestland and cultivated land exhibited a decreasing trend from 2000 to 2010. The HQ indices of cultivated land and forestland improved and exhibited an increasing trend from 2010 to 2018, whereas those of grassland and water decreased. With the increase in population, the demand for land is likely to increase accordingly, and more forestland and grassland will be cleared for cultivation, as well as the conversion of large areas of cultivated land to construction land. Therefore, HQ indices of various LULC are affected by human activities. However, the implementation of active interventions, such as restoration of forestland and grassland and relevant environmental conversation activities, has led to a decline in habitat degradation of land-use types disturbed by anthropogenic factors has declined and improved HQ. The HQ index of forestland over the 1990–2018 period remained above 0.73, which could be because the forestland in Guizhou Province is mainly distributed in high altitude and sloping areas. Furthermore, mountainous areas are not conducive to activities, such as cultivation and construction land development. Although the HQ index of forestland remained high during the 1990–2018 period, the total forestland area decreased, which is attributed to the considerable degradation of forestland through its conversion to cultivated and construction land. However, topographic conditions of forestland limit human activities and the high vegetation cover makes it more resistant to disturbances. HQ has been maintained at a high level due to the implementation of various ecological conservation activities and restoration practices.

### Factors influencing spatial variations in HQ

#### Single factor analysis

GeoDetector analysis results (Table 8) revealed differences in the interpretation abilities (q-values) of the various factors influencing variations in HQ. The order of the q-values was land-use type (X9) > NDVI (X6) > slope (X2) > GDP > NPP (X5) > temperature (X3) > rainfall (X4) > elevation (X1) > population density (X7). Therefore, land-use type was identified as a key factor influencing variations in HQ (q-value of 0.264), followed by NDVI (q-value of 0.106). Slope, temperature, rainfall, population density, elevation, and GDP had weak interpretation ability for spatial variations in HQ; however, the factors should be taken into consideration. Overall, natural factors (land-use type, NDVI, and NPP) significantly influenced the spatial distribution patterns of HQ and their q-values were greater than those of topographic (slope, elevation), socioeconomic (GDP, population density), and meteorological (precipitation, temperature) factors.

**Table 8:** q-values of factors influencing spatial variations in HQ.

Driving factor	X1	X2	X3	X4	X5	X6	X7	X8	X9
Driving force (q)	0.018	0.085	0.034	0.025	0.041	0.106	0.012	0.051	0.264

#### Analysis of interactions between two factors

The interaction detector of the GeoDetector was used to test the explanatory power of two-factor interactions on HQ and the results (Table 9) indicated that the q-values of two-factor interactions were greater than the q-value of a single factor. The interactions exhibited a two-factor enhancement or non-linear enhancement. The most significant interactive effect on the spatial variation in HQ was that of land-use type ∩ NDVI (0.309), followed by that of land-use type ∩ slope (0.300). The most dominant factor interaction was observed when a dominant single factor (land-use type) was combined with another factor, suggesting that different land-use types determine the distribution patterns of ecosystem types. The interaction of NDVI with precipitation, temperature, and slope exhibited considerable interpretation ability, indicating that natural factors, such as temperature, precipitation, and slope affect changes in land-use type and in turn, HQ to a certain extent. Although the q-values of single factors, GDP and population density were low, their interaction with other factors exceeded the interpretation ability of single factors influencing the spatial differentiation of HQ, which highlights the need to take such factors into consideration. Urbanization and population growth will lead to rapid expansion of construction land and consequently increase ecological pressure on the surrounding habitats. Therefore, the spatial patterns of towns and cities should be optimized to improve the local HQ through the implementation of ecological restoration projects.

**Table 9:** The results of interactive detection.

Interaction	Influence	Interaction	Influence
X1 ∩ X2 (0.105)	Enhance, nonlinear-	X3 ∩ X7 (0.045)	Enhance, bi-
X1 ∩ X3 (0.043)	Enhance, bi-	X3 ∩ X8 (0.082)	Enhance, bi-
X1 ∩ X4 (0.058)	Enhance, nonlinear-	X3 ∩ X9 (0.289)	Enhance, bi-
X1 ∩ X5 (0.061)	Enhance, nonlinear-	X4 ∩ X5 (0.064)	Enhance, bi-
X1 ∩ X6 (0.124)	Enhance, bi-	X4 ∩ X6 (0.129)	Enhance, bi-
X1 ∩ X7 (0.029)	Enhance, bi-	X4 ∩ X7 (0.037)	Enhance, bi-
X1 ∩ X8 (0.067)	Enhance, bi-	X4 ∩ X8 (0.073)	Enhance, bi-
X1 ∩ X9 (0.272)	Enhance, bi-	X4 ∩ X9 (0.282)	Enhance, bi-
X2 ∩ X3 (0.112)	Enhance, bi-	X5 ∩ X6 (0.126)	Enhance, bi-
X2 ∩ X4 (0.113)	Enhance, nonlinear-	X5 ∩ X7 (0.053)	Enhance, bi-
X2 ∩ X5 (0.118)	Enhance, bi-	X5 ∩ X8 (0.088)	Enhance, bi-
X2 ∩ X6 (0.161)	Enhance,bi-	X5 ∩ X9 (0.278)	Enhance, bi-
X2 ∩ X7 (0.096)	Enhance,bi-	X6 ∩ X7 (0.109)	Enhance, bi-
X2 ∩ X8 (0.120)	Enhance, bi-	X6 ∩ X8 (0.125)	Enhance, bi-
X2 ∩ X9 (0.300)	Enhance, bi-	X6 ∩ X9 (0.309)	Enhance, bi-
X3 ∩ X4 (0.070)	Enhance, nonlinear-	X7 ∩ X8 (0.054)	Enhance, bi-
X3 ∩ X5 (0.075)	Enhance, bi-	X7 ∩ X9 (0.269)	Enhance, bi-
X3 ∩ X6 (0.145)	Enhance, nonlinear-	X8 ∩ X9 (0.290)	Enhance, bi-

Enhance, bi-: means that the interaction between the two factors is a two-factor enhancement, Enhance nonlinear: means a non-linear enhancement.

## Discussion

The present study assessed the spatio-temporal changes in HQ of Guizhou Province from 1990 to 2018 and factors influencing HQ using the HQ module of the InVEST model and GeoDetector. The research has significant implications for biodiversity conservation and the construction of ecological civilization in karst regions.

The essence of land-use transformation is to change the form of land-use during socio-economic development. The primary cause of land-use transformation is the change in land-use types leaded by human activities on natural ecosystems^52^. The results of the present study have shown that land-use has changed considerably over the last few decades, with cultivated land, grassland, and forestland being the predominant land-use types in Guizhou Province. The results of the present study revealed that cultivated land and forestland areas decreased during the 1990–2018 period, while construction land area increased with increasing economic development and urban expansion, which explains the decline in HQ in Guizhou Province. Moreover, the development of rural infrastructure has accelerated due to the revitalization of the countryside and tourism, in turn, promoting sustainable development of the regions in Guizhou Province. These projects have provided favorable conditions for addressing poverty in rural regions, although they have also had considerable negative impacts on the ecosystem, leading to a reduction in HQ. The average HQ index of Guizhou Province during the 1990–2018 period decreased slightly and was characterized by an initial decrease, followed by an increase, and then a decrease. However, the implementation of a range of restoration and protection measures, such as returning cultivated land to forestland and grassland and rocky desertification treatment, have substantially enhanced the ecological environment in Guizhou Province. The increase in population, economic development, and urbanization during the 1990–2018 period has led to an increase in construction land area by nearly four times. The expansion of construction land has created new sources of threats, disturbed and destroyed the surrounding habitats, leading to a decline in HQ.

The dominant factor influencing HQ during the study period was land-use type, as determined by single factor detection. Land-use type is a key factor determining regional HQ, and land-use types determine the distribution patterns of various ecosystems, especially the occupation of ecological spaces by construction land, which accelerates landscape fragmentation and leads to a decline in HQ. Followed by NDVI index, which exhibits a strong influence on HQ, and is a key parameter that reflects vegetation growth status and cover. NDVI is closely associated with HQ^53^. According to the interaction detection results, n atural and socio-economic factors had a substantial effect on the spatio-temporal variations in HQ of the study area. The interaction between factors enhanced the effect on HQ, indicating that two-factor interaction effect was greater than that of a single factor. The interactive effect between land-use and other factors was considerably stronger than that between other factors, indicating that land-use is a major factor influencing HQ changes.

The InVEST-HQ model was used in this study to provide a feasible method for the quantification of HQ and visualization of the calculated results. However, our study had some limitations. First, the results of regional-scale analysis can only be used as a reference for the relationship between LULC changes and variations in HQ in Guizhou Province. However, specific conservation measures need to be studied on a more comprehensive scale. Second, the InVEST-HQ model requires several parameters, and the relevant parameters used in this study were obtained from the model manual, previous studies, and expert experience that are associated with a certain degree of subjectivity in the parameter settings, which could lead to uncertainty in the evaluated results. Final, inconsistencies in data accuracy, which result in certain deviations in factor extraction when using fishnet sampling, may have led to deviations in the driving force results obtained using the GeoDetector model.

## Conclusions

The present study investigated the spatio-temporal variations in HQ and factors influencing HQ changes in Guizhou Province, using the InVEST-HQ model and GeoDetector. The main conclusions are as follows:

1. The main land-use types in Guizhou Province during the 1990–2018 period were forestland, cultivated land, and grassland. Cultivated land and forestland decreased by − 98.27% and − 98.37%, respectively, while grassland, construction land, and water increased by 100.41%, 398.71%, and 264.21%, respectively; unused land was relatively stable.
2. The average HQ index of Guizhou Province during the 1990–2018 period initially decreased, then increased, and eventually decreased. The spatial distribution of hot and cold spots associated with HQ showed a pattern of "high in the south and east, low in the central and west, and high and low in the north".
3. Single and interactive factor detection using the GeoDetector showed that the major factor influencing HQ changes was LULC, followed by natural factors, such as NDVI and slope. Socio-economic factors such as population density and GDP were minor factors influencing HQ changes (Supplementary Information).

## Supplementary Information

Supplementary Information 1.

Supplementary Information 2.

Supplementary Information 3.

Supplementary Information 4.

Supplementary Information 5.

## Data Availability

The datasets used and/or analysed during the current study available from the corresponding author on reasonable request.
