# Author Correction: Spatio-temporal evolution and driving forces of habitat quality in Guizhou Province

**DOI:** 10.1038/s41598-023-35348-5

**Published:** 2023-05-22

**Authors:** Bo Xie, Mingming Zhang

**Affiliations:** 1 College of Forestry, Guizhou University, Guiyang, 550025 Guizhou China; 2 Research Center for Biodiversity and Nature Conservation, Guizhou University, Guiyang, China

Correction to: *Scientific Reports* 10.1038/s41598-023-33903-8, published online 27 April 2023

The original version of this Article contained an error in the Abstract.

“High habitat quality areas were mainly located in the western part of Guizhou Province, whereas low habitat quality areas were located in the central region.”

now reads:

“The most significant improvement of habitat quality was in the western region, whereas the most significant decline of habitat quality was in the central region.”

The original Article has been corrected.

